# Progress in Medicine

**Published:** 1898-12-10

**Authors:** 


### Progress in Medicine.

### DIPHTHERIA.

(*Continued from page* 163)

Johnson,[5] superintendent of the Glasgow Fever Hospital, in his last report, states that 25·7 per cent. of the cases sent into the hospital as diphtheria turned out not to be that complaint. What is diphtheria? Till recently it was generally admitted that if the Klebs-Löffler bacillus was proved to be present in the culture from any individual's throat, that person was the subject of diphtheria, whether the symptoms were marked or so slight as to be unnoticeable. More recent observations have brought home to our minds the fact that such a view is no longer tenable. F. L. Wachenheim[6] reviews the whole question in an article on the clinical relations of the diphtheria bacillus. He recalls the two views as to the nature of diphtheria current prior to the discovery of the bacillus, viz., those of Bretonneau and Virchow. Bretonneau claimed a common origin for croup and malignant angina, declaring them both due to the same infecting agent, which caused a superficial fibrinous deposit to form on the affected mucous membranes. Any necrotic changes he attributed to secondary influences, and he rightly excluded scarlatinal angina from genuine diphtheria. He based this distinction on the firm adhesion of the scarlatinal membrane, its limitation to the fauces and absence from the larynx, and its failure to confer immunity against true diphtheria. Virchow, arguing from the histological standpoint, defined "diphtheritic inflammation" as one attended with the deposit of a fibrinous pseudo-membrane and necrosis of the underlying tissues. Fibrinous exudation without necrosis he termed "croupous inflammation." He spoke of diphtheria caused by chemical agents, diphtheritic colitis in dysentery, and wound diphtheria. Wagner showed that croupous and diphtheritic inflammation differed merely in degree, and might be dependent on the same ætiological factor.

The author gives a brief morphological outline of tonsillar inflammations, as they present themselves clinically, viz.: (1) Catarrhal angina; (2) the same, with white dots occupying the lacunæ of the tonsil superadded (punctate croupous tonsilitis); (3) the same, but with the spots larger and spread over the surface of the gland, dipping down into the lacunæ, but frequently not starting from them (disseminated croupous tonsilitis); (4) when the patches of the third form fuse we obtain the croupous angina of Virchow; (5) diphtheritic angina of Virchow; (6) gangrenous tonsilitis, differing from the fifth class only in the depth of the necrotic process.

According to the old standard membrane is the one characteristic feature, and (excluding local traumatisms) it determined the diagnosis of diphtheria.

Löffler and later observers (Escherich, Kossel, and Baginsky) found the Löffler bacillus in from 92 to 97 per cent. of cases of primary diphtherias. In the absence of this micro-organism, various cocci, chiefly the streptococcus pyogenes and the so-called pseudo-diphtheria bacillus, appeared. Concerning the pseudo-diphtheria bacillus, two views seem about equally represented: the one, that it is an attenuated Löffler bacillus; the other, that it is a distinct germ. Roux and Yersin and C. Fraenkel accept the former; Escherich, Löffler, and Park the latter. Spronck[7] claims to distinguish between them by the serum test, but C. Fraenkel[8] disposes of this method as fallacious. Max Neisser's recently announced staining reactions seem likely to meet the same fate. Koplik[9] has demonstrated the "pseudo" bacillus replacing the Löffler bacillus in two cases. A thorough survey of the literature will persuade the impartial clinician that the matter is still decidedly *sub judice*.

Proceeding to the streptococcus cases, we must remember that in some instances the Löffler bacillus may escape observation by appearing late or disappearing early. There seems to be little doubt of the infectiousness of streptococcus throat affections. Park and Beebe[10] concede a mortality of 4 per cent. from laryngeal and pneumonic complications, and we must attribute to the streptococcus such secondary lesions as otitis media and suppurative adenitis. Bernheim and Roux and Yersin have proved the high virulence of a mixed Löffler bacillus and streptococcus infection. It seems to Wachenheim, therefore, a grave error to regard these cases as of minor importance.

The diphtheria bacillus was demonstrated by Koplik in a few cases of punctuate and many cases of disseminated croupous angina, while Park has also referred to the import of the presence of diphtheria bacilli in a comparatively few lacunar tonsilitis cases. But the bacillus has also been found in normal throats; especially is it frequently found in the throats of nurses and others who have been exposed to infection. Of 330 persons who gave no history of exposure, Park and Beebe[11] found Löffler bacilli in 24 cases, eight times fully virulent. Further, the bacillus has been shown by several authorities to be the cause of fibrinous rhinitis.

A most interesting subject is the persistence of the bacillus in the throats of convalescents; it must influence greatly the matter of isolation. Escherich[12] found fully virulent bacilli after five weeks; Park after seven weeks; Belfonti[13] after seven months. Tobiesen,[14] however, shows that they rarely, if ever, transmit the disease. It is notable that the germ is most persistent in persons affected with chronic inflammation of the air passages. Wachenheim then proceeds to give an instructive epitome of cases collected by him. Sixteen cases presented typical membranes; in ten of these the Löffler bacillus was demonstrated. Of 14 cases of lacunar and catarrhal angina only one presented Löffler bacilli. Wachenheim states that he sees no reason to assume, from the mere finding of the characteristic germ, that a simple lacunar angina is a diphtheria in any clinical sense. The absolutely excellent prognosis as to recovery, the freedom from paralytic sequelæ, are alone almost sufficient evidence. If, moreover, the bacillus is to determine our diagnosis, how about normal individuals who happen to be carrying it in their fauces? Is it rational to isolate a person who has had diphtheria for seven months on purely bacteriological grounds? The only really scientific test, that of virulence, cannot, from lack of time, be made by any health office in all cases presented to it. We have quoted this timely and very scientific contribution very fully, and would further emphasise the importance of the author's concluding remarks: "Thus, our diagnostic procedure is practically restricted almost to that of the great clinician, Bretonneau. Our bacteriological results are at best only confirmatory, and we are required in practice to manage our cases from the outset from a purely clinical standpoint. In the realm of medicine there is at present a tendency to supplant bedside observation by laboratory research. In this paper I have sought to impress the view that in diphtheria our bacteriological studies should subserve our clinical methods, not control them." The vitality of the diphtheria bacillus has arrested the attention of many others.

**Diphtheritic Rhinitis.**—In support of Wachenheim's concluding remarks, we may refer to C. Todd's[15] remarks on an external rhinitis due to the diphtheria bacillus appearing in children convalescent from scarlet fever.

*Clinical History.*—The first sign of anything abnormal is a slight redness of the posterior margin of one or both nostrils, usually beginning at the inner or outer angle and at the muco-cutaneous junction. The redness becomes more intense, and ultimately a moist, granular-looking, raw surface results; this surface bleeds easily, and is often covered by a crust which may almost or completely block up the nostril. This is more commonly the case in younger children, who scratch their nostrils and so cause bleeding. There is never any formation of membrane, and the process does not appear to extend backwards into the nasal cavity, but in many cases it spreads down to the upper lip in the form of an eczematous area, apparently caused by the infective discharge. This discharge is usually slight, and not uncommonly absent. The nostrils remain in this granular condition for a variable time—from one to four or five weeks—and then gradually resume their normal condition. Fifty-one cases occurred amongst 365 children—almost 14 per cent., and in each of the 51 the diphtheria bacillus was found.

Todd summarises his observations thus: (1) Children convalescent from scarlet fever in hospital are very liable to a certain form of external rhinitis, often accompanied by the formation of secondary pustules on various parts of the body; (2) this rhinitis, though not membranous, is associated with the presence of the Klebs-Löffler bacillus in the nostrils, this organism being absent from the fauces; (3) it is contagious as such, but has not been observed to give rise to faucial or laryngeal diphtheria; (4) it is unaccompanied by rise of temperature, albuminuria, or marked glandular enlargement; (5) it appears to be limited to children under 13 years of age, and has been most frequently observed at the ages of three and four years. The fact that the bacillus, though present in the nostrils in large numbers, and causing a local lesion, does not give rise to any constitutional symptoms or to faucial or laryngeal diphtheria, suggests that its virulence is modified to a remarkable degree. It is virulent to guinea pigs when inoculated subcutaneously, but this is no criterion of its virulence to the human being, as was shown by Dr. Klein in the case of diphtheria bacilli taken from the fauces of patients suffering from diphtheria. Why the bacillus limits itself to the nostrils is very hard to see. It appears not improbable that under certain conditions this feebly virulent bacillus may acquire a higher degree of virulence.

[5] Treatment, Nov. 10, 1898. [6] New York Med. Jour., June 18, 1898. [7] Deut. Med. Woch., No. 36, 1895. [8] Hygienish. Runds chau., No. 6. [9] New York Med. Jour., Mar. 10, 1898. [10] Med. Rec., Sept. 29, 1894. [11] Loc. cit. [12] Epidemische Diphtheriè. [13] Rif. Med., Mar. 23, 1894. [14] Centralbl. f. Bakt., xii. [15] Lancet, May 28, 1898.

